# Hyperpigmentation Results in Aberrant Immune Development in Silky Fowl (*Gallus gallus domesticus* Brisson)

**DOI:** 10.1371/journal.pone.0125686

**Published:** 2015-06-05

**Authors:** Deping Han, Shuxiang Wang, Yanxin Hu, Yuanyuan Zhang, Xianggui Dong, Zu Yang, Jiankui Wang, Junying Li, Xuemei Deng

**Affiliations:** 1 National Engineering Laboratory for Animal Breeding and Key Laboratory of Animal Genetics, Breeding, and Reproduction of the Ministry of Agriculture, China Agricultural University, Beijing, 100193, China; 2 College of Veterinary Medicine, China Agricultural University, Beijing, 100193, China; University of Alabama at Birmingham, UNITED STATES

## Abstract

The Silky Fowl (SF) is known for its special phenotypes and atypical distribution of melanocytes among internal organs. Although the genes associated with melanocyte migration have been investigated substantially, there is little information on the postnatal distribution of melanocytes in inner organs and the effect of hyperpigmentation on the development of SF. Here, we analyzed melanocyte distribution in 26 tissues or organs on postnatal day 1 and weeks 2, 3, 4, 6, 10, and 23. Except for the liver, pancreas, pituitary gland, and adrenal gland, melanocytes were distributed throughout the body, primarily around blood vessels. Interaction between melanocytes and the tissue cells was observed, and melanin was transported by filopodia delivery through engulfed and internalized membrane-encapsulated melanosomes. SFs less than 10 weeks old have lower indices of spleen, thymus, and bursa of Fabricius than White Leghorns (WLs). The expression levels of interferon-γ and interlukin-4 genes in the spleen, and serum antibody levels against H5N1 and infectious bursal disease virus were lower in SF than in WL. We also found immune organ developmental difference between Black-boned and non-Black- boned chickens from SFs and WLs hybrid F2 population. However, degeneration of the thymus and bursa of Fabricius occurred later in SF than in WL after sexual maturity. Analysis of apoptotic cells and apoptosis-associated Bax and Bcl-2 proteins indicated that apoptosis is involved in degeneration of the thymus and bursa of Fabricius. Therefore, these results suggest that hyperpigmentation in SF may have a close relationship with immune development in SF, which can provide an important animal model to investigate the roles of melanocyte.

## Introduction

The Silky Fowl (SF) is a natural mutant breed in China with unique morphological features such as fluffy head feathers, rose comb, blue earlobes, silky feathers, black skin, hair-like leg feathers, and five toes. Besides the skin, hyperpigmentation has been observed in the internal organs of SF. This has drawn the attention of numerous researchers interested in investigating the molecular mechanism of melanocyte development [1–4]. The migratory path of melanoblasts and premelanocytes and the identities of the genes that are involved in migration during early embryogenesis are known [5–10]; however, no reports have addressed the distribution or function of melanocytes in different tissues from hatching to reproductive maturity.

Melanocytes protect the skin from ultraviolet radiation by shielding DNA from damage [11, 12]. Moreover, perivascular-resident macrophage-like melanocytes maintain the integrity of the interstitial fluid-blood barrier by regulating the expression of several tight junction-associated proteins [13]. Inflammation caused by trauma attracts melanocytes and melanoblasts to the site of injury after initial recruitment of cells of the innate immune system, suggesting that cytokines produced by immune cells induce melanocyte functions that mediate wound repair [14]. Melanin and other associated products contribute to the regulation of immune response, resistance to fatigue, and protection against oxidative stress in SF [15–18]. The role of melanin in these processes is intriguing, but the underlying mechanism remains to be elucidated. Comprehensive understanding of the mechanism of benign hyperpigmentation may facilitate investigations of the functions of melanocytes during the development of SF and may help understand the pathogenesis of melanoma in mammals.

A few studies have analyzed the effects of hyperpigmentation in inner organs that affect the development of SF. In our previous work, we found that genes involved in the innate and adoptive immune responses are up and down regulated, respectively, during embryonic development on days 3, 3.5, 4, and 4.5 [19]. In the present study, we determined the histological distribution of melanocytes and analyzed the populations of immune cells and cytokine gene expression in immune organs during development in SF, White Leghorn (WL), and the hybrid F2 generation birds.

## Materials and Methods

### Animals

We studied 42 SFs and 42 WLs (equal numbers of females and males) aged 1 day and 2, 3, 4, 6, 10, and 23 weeks; and 6 Black-boned and 6 non-Black-boned chicken from the hybrid F2 generation (equal numbers of females and males) aged 6 weeks. The chickens were obtained from the China Agricultural University’s animal farm. The Beijing Municipal Committee of Animal Management and The Ethics Committee of China Agricultural University approved the protocols for animal use and experimentation.

### Organ indices and samples

The chickens were sacrificed by severing the jugular veins after anesthesia, bled for 3–5 min, and then dissected. The weights of the spleen, thymus, and bursa of Fabricius were determined, and the organ indices were calculated using the following formula: organ index = organ weight/body weight × 100%. All tissues were sampled in duplicate; one sample was fixed in 4% paraformaldehyde for paraffin sectioning, and the other was stored in liquid nitrogen for cryosectioning.

### 3, 4-Dihydroxy-l-phenylalanine (DOPA) staining

The tissues were embedded in OCT (Opti-mum Cutting Temperature compound, Leica, Germany) to prepare 7-μm serial sections. The sections were stained according to the method described by Rui [20], with the following modifications: frozen sections were equilibrated to room temperature, rinsed in distilled water, and incubated in DOPA buffer containing 1.104 g of DOPA (Sigma-Aldrich, Germany) in 1 L of 0.01 mol/L phosphate-buffered saline (PBS) for 30 min at 37°C. The sections were then incubated in fresh DOPA buffer for 30 min at 37°C or until the color of the buffer darkened. After counterstaining with 1% Nuclear Fast Red buffer for 10 min at room temperature, the sections were dehydrated in 100% alcohol and xylene, and then they were mounted for observation using a light microscope. Stained cells exhibited black cytoplasm. Control staining reactions were performed using PBS instead of DOPA.

### Hematoxylin and eosin (H&E) staining

Tissues were fixed for at least 24 h, trimmed, dehydrated using alcohols, embedded in paraffin, and cut into 5-μm serial sections. The sections were dewaxed, rehydrated, and incubated in hematoxylin (Zhongshan Golden Bridge Biotechnology Co, Beijing, China) for 5 min. The sections were rinsed with distilled water, followed by 1% HCl in 75% alcohol for 20 s, and then incubated in PBS for 5 min. The sections were incubated in 70% and 80% alcohol for 2 min each and then stained with eosin (Zhongshan Golden Bridge Biotechnology) for 30 s. After destaining in 95% alcohol for 1 min, the sections were dehydrated in 100% alcohol and xylene, and then they were mounted for light microscopy observations to investigate the histological distributions of melanin.

### Toluidine blue staining

Mast cells were analyzed using an improved toluidine blue staining method [21]. Briefly, the paraffin sections were dewaxed, rehydrated, and immersed in 0.8% toluidine blue (Sigma, Shanghai, China) for 15 s. The sections were rinsed with distilled water, placed in 95% alcohol until the mast cells appeared deep reddish-purple, dehydrated, and then mounted. The distribution of mast cells in the tissues was observed under a light microscope.

### Ultrastructural observations

The tissues were trimmed (1–2 mm^-3^) and fixed overnight at 4°C in 2.5% glutaraldehyde-2%paraformaldehyde in 5 mM sodium phosphate (pH 7.4)-buffered 0.9% (w/v) saline, gently rinsed in 0.1 M sodium phosphate buffer (PB; pH 7.2), post-fixed with 1% osmium tetroxide in 0.1 M PB and rinsed thoroughly with the same buffer again. The tissues were then dehydrated through a series of ascending grades of acetone at room temperature, embedded in SuperResin, and polymerized at 60°C for 2 days. Subsequently, they were cut into 70 nm-thick sections on a Leica ultramicrotome (Austria), and stained with uranylacetate for 8 min, followed by lead citrate for 4 min. The ultrathin sections were examined under a transmission electron microscope (JEM-1230, Japan) to observe the ultrastructural features of the cells and to elucidate the relationships among the cells in the microenvironment.

### Immunofluorescence assay

Frozen sections were equilibrated to room temperature, rinsed in distilled water, and incubated in 3% hydrogen peroxide solution for 15 min at room temperature. The sections were washed with distilled water and blocked with 1% bovine serum albumin for 20 min at room temperature. After discarding the blocking solution, the sections were incubated with fluorescein isothiocyanate (FITC)-labeled mouse anti-chicken CD3^+^ antibodies (1:500, Southern Biotech, Alabama, USA) overnight at 4°C in a humidified chamber. After rinsing with PBS, the sections were observed using a fluorescence microscope. In the negative control, the primary antibody was replaced with PBS. The numbers of CD3^+^ cells were counted in 10 high-power fields at ×200 magnifications, and the means were calculated. Sampling of the sections was unbiased; all the samples were coded and the examinations were performed by a single investigator.

### Immunohistochemistry

Frozen sections were washed with distilled water, incubated in 3% hydrogen peroxide solution for 15 min at room temperature, washed with distilled water again, and blocked with 1% bovine serum albumin for 20 min at room temperature. The sections were incubated with primary mouse anti-chicken Bu-1 (Santa Cruz, Texas, USA, 1:100), rabbit anti-Bax (Santa Cruz, Texas, USA, 1:200), and mouse anti-Bcl-2 (BD Biosciences, California, USA, 1:200) antibodies overnight at 4°C. After rinsing with PBS, the sections were treated with goat anti-mouse and anti-rabbit immunoglobulin G (IgG) conjugated to horseradish peroxidase (Zhongshan Golden Bridge Biotechnology, Beijing, China) at room temperature for 1 h. After treating the sections with chromogen diaminobenzidine (Zhongshan Golden Bridge Biotechnology, Beijing, China) for 10 min at room temperature in the dark, the sections were counterstained with hematoxylin. The primary antibodies were replaced with PBS in the negative controls. The densities of Bu-1 in the bursa were identified in 10 high-power lymph nodes using Image-Pro plus 6.0, and the means were calculated. The intensities of Bax and Bcl-2 in 10 high-power fields for each sample were determined using Image-Pro plus 6.0, and the means were calculated. Sampling of the sections was unbiased; all the samples were coded and the examinations were performed by a single investigator.

### Quantitative polymerase chain reaction (Q-PCR)

Total RNA was isolated from the spleen samples using TRIzol reagent, according to the manufacturer’s protocol (Invitrogen, USA). The RNA was purified using an RNeasy mini kit (Qiagen, USA). The RNA quality and purity were determined using a NanoDrop ND-1000 spectrophotometer at 260/280 nm (NanoDrop Technologies, USA). Total RNA (0.5 μg) was transcribed into cDNA using the PrimeScript RT reagent Kit (Takara, Japan), according to the manufacturer’s instructions. Primers were designed using Primer Express 2.0 (Applied Biosystems, USA) and synthesized (Sangon Biotech, Beijing, China). The primer sequences are listed in Table 1. The expression levels of interferon-γ (*IFN-γ*), interlukin-4 (*IL-4*), and gallinacin-7 (*GAL-7*) in the spleens were quantified by quantitative PCR using the SYBR Green Real-time PCR Master Mix (Takara, Japan). The cycling parameters used for quantitative PCR amplification were as follows: initial heat-denaturation at 95°C for 4 min, followed by 40 cycles at 95°C for 30 s, 58°C for 30 s, and 72°C for 30 s, and extension for 5 min at 72°C finally. A melt curve analysis was performed to exclude genomic DNA contamination and to confirm primer specificities. Gene expression was normalized using the 2^—△CT^ method with glyceraldehyde 3-phosphate dehydrogenase (*GAPDH*) as the internal standard. Data were collected from three biological duplicates, and each biological duplicate was controlled in three technical replicates. *P* values less than 0.05 were considered statistically significant.

**Table 1:** The primers were used to detect the expressions of *IFN-γ*, *IL-4* and *GAL-7* in the spleens.

Genes	Primers	
***IFN-γ***	Forward	5' GTGAAGAAGGTGAAAGATATCATGGA 3'
	Reverse	5' GCTTTGCGCTGGATTCTCA 3'
***IL-4***	Forward	5' AACATGCGTCAGCTCCTGAAT 3'
	Reverse	5' TCTGCTAGGAACTTCTCCATTGAA 3'
***GAL-7***	Forward	5' TGGAGAAGGGAGACAGAAGG 3'
	Reverse	5' GGACAGACAGCAGCAGGTAA 3'
***GAPDH***	Forward	5' AAAGTCCAAGTGGTGGCCATC 3'
	Reverse	5' TTTCCCGTTCTCAGCCTTGAC 3'

### Immunization and antibody detection

**Fig 1:**
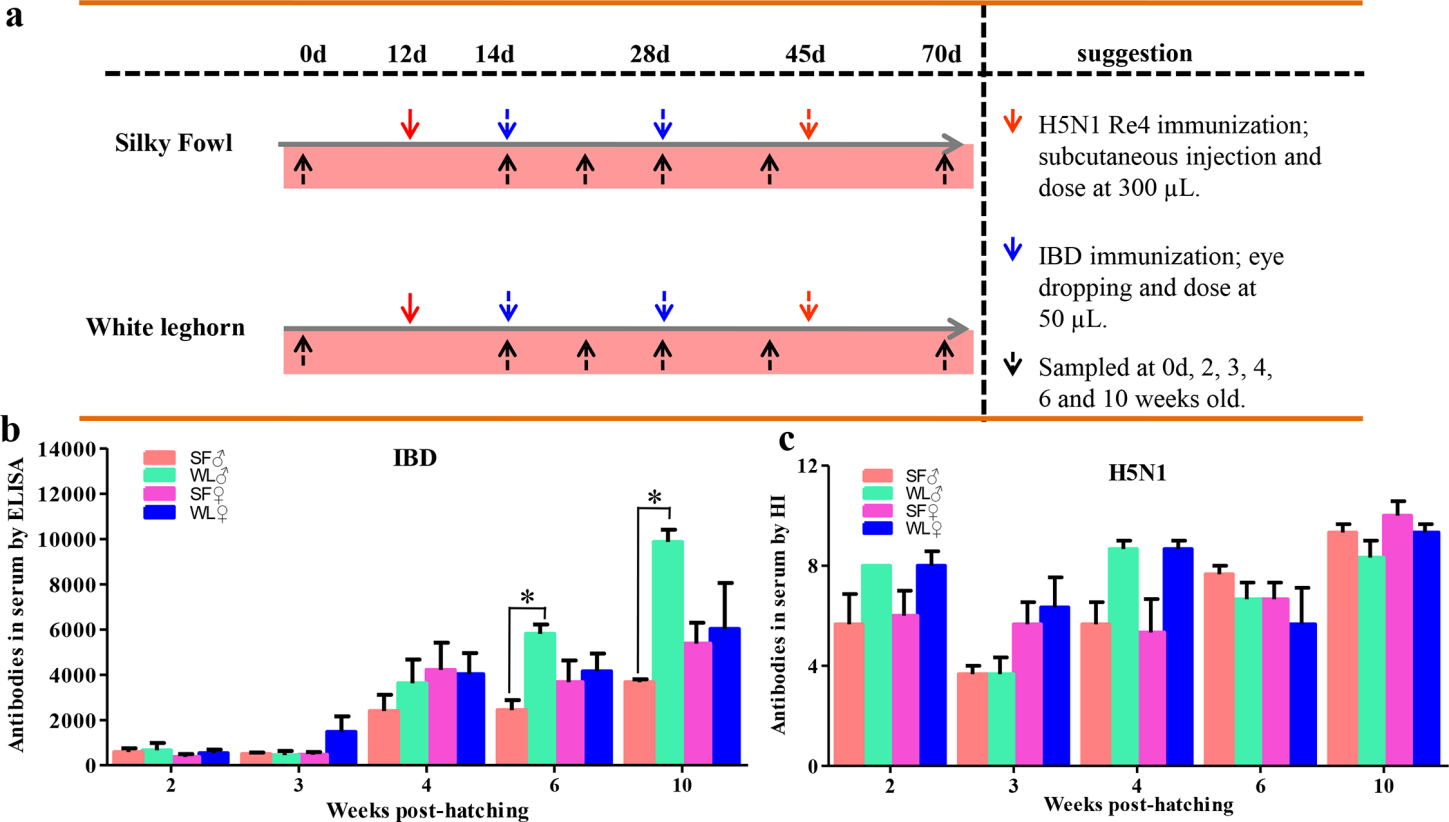
Immunization and antibody detection in the serum of SF and WL. Vaccines, injection routes, doses, and sample times are listed (a). Lower antibody levels against H5N1 Re4 were detected in the serum of SF, and obvious differences were calculated between SF and WL in females at 6 and 10 weeks of age (b). Antibody level against infectious bursal disease virus (IBDV) was low in the serum of SF, but no difference was detected between the SF and WL (c). *P < 0.05.

Additional fifty SFs and fifty WLs were immunized with avian influenza virus (H5N1 Re4) and moderate-virulence infectious bursal disease virus (IBDV) vaccines (China Institute of Veterinary Drug Control, Beijing, China). Blood was sampled from the wing veins at different times post-immunization, and the serum was centrifuged. We detected the antibodies against H5N1 Re4 and IBDV in the serum by hemagglutinin inhibition assay and enzyme-linked immunosorbent assay, respectively. Additional details of the immunization and sample collection protocol are shown in Fig 1.

### TUNEL assays

The presence of apoptotic cells was determined using the *In Situ* Cell Death Detection Kit (Roche, Philadelphia, PA). Paraffin-embedded sections of spleen, thymus, and bursa of Fabricius were dewaxed, rehydrated according to standard procedures, and immersed in a plastic jar containing 200 mL of citrate buffer (0.1 M, pH 6.0). The slides were subjected to microwave irradiation (750 W [high]) for 1 min, cooled rapidly by immediately adding 80 mL of double-distilled water, and then transferred into PBS. The slides were immersed for 30 min in Tris-HCl (0.1 M, pH 7.5, containing 3% BSA and 20% normal bovine serum) and then rinsed twice with PBS. The preceding steps were performed at 20–25°C. The TUNEL reaction mixture was added to the slides, which were then incubated at 37°C in a humidified atmosphere in the dark for 60 min. The slides were rinsed three times in PBS for 5 min each. Samples were analyzed in a drop of PBS using a fluorescence microscope with excitation and detection wavelengths of 450–500 nm and 515–565 nm (green), respectively. The TUNEL reaction mixture was replaced with label solution in the negative control, and the number of positive cells was counted as described previously in the part of immunofluorescence assay for CD3^+^ cells.

### Statistical analysis

Data were expressed as means ± standard error (SE). The significance of the variability among different breeds or groups was determined using one-way analysis of variance (ANOVA), as implemented in the SPSS software suite (version 12.0; SPSS Taiwan Corp., Taiwan). A *P*-value of <0.05 was considered statistically significant.

## Results

### Hyperpigmentation in SF

**Fig 2:**
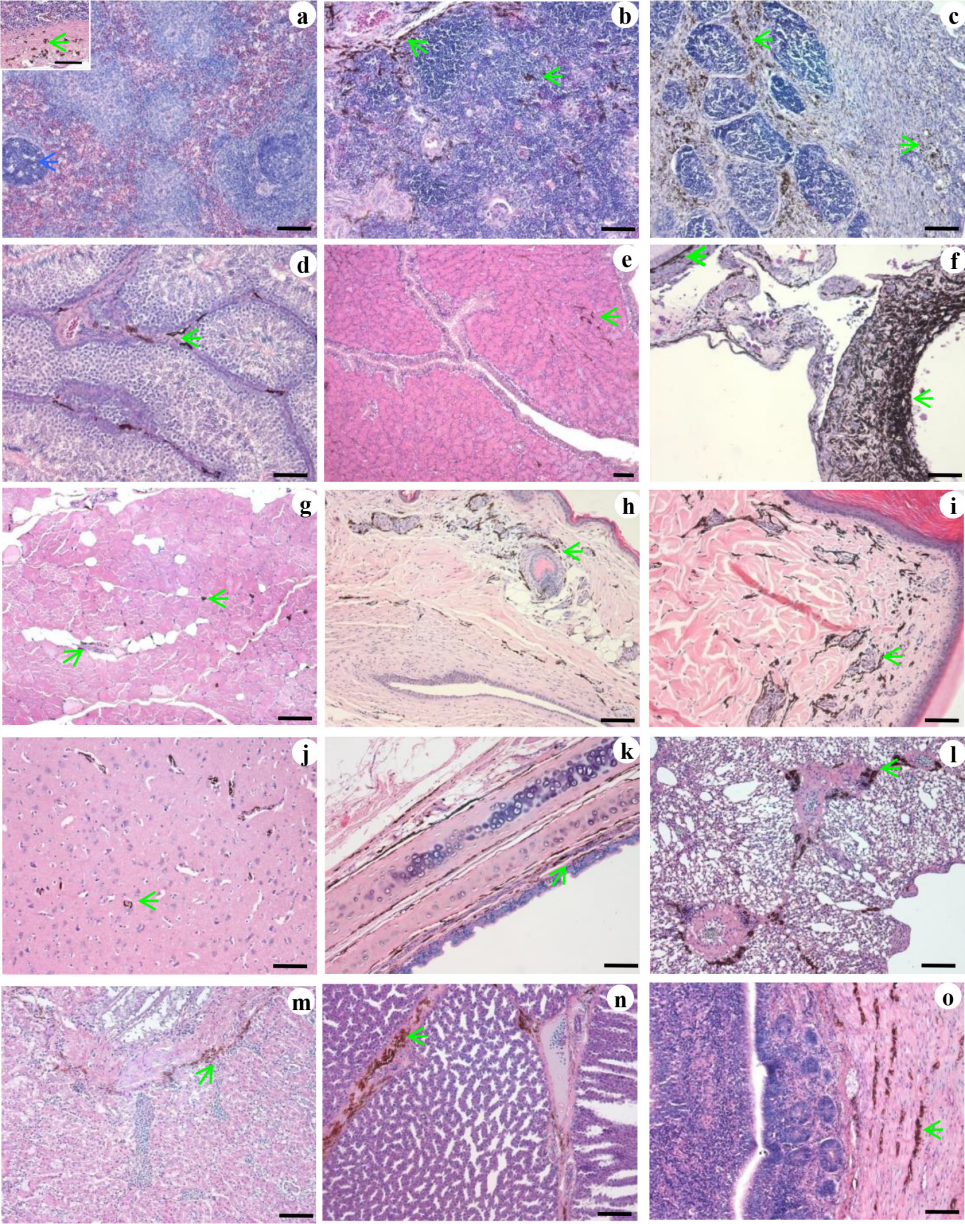
Tissue distribution of melanocytes in Silky Fowl (SF). Spleen capsule and parenchyma (a). Thymus (b). Bursa of Fabricius (c). Testis (d). Oviduct (e). Ovary (f). Leg muscle (g). Dorsal skin (h). Leg skin (i). Brain (j). Trachea (k). Lung (l). Kidney (m). Stomach (n). Intestine (o). Melanocyte: green arrow. Lymph node: blue arrow. Hematoxylin and eosin (H&E) stain. Scale bar = 100 μm.

**Fig 3:**
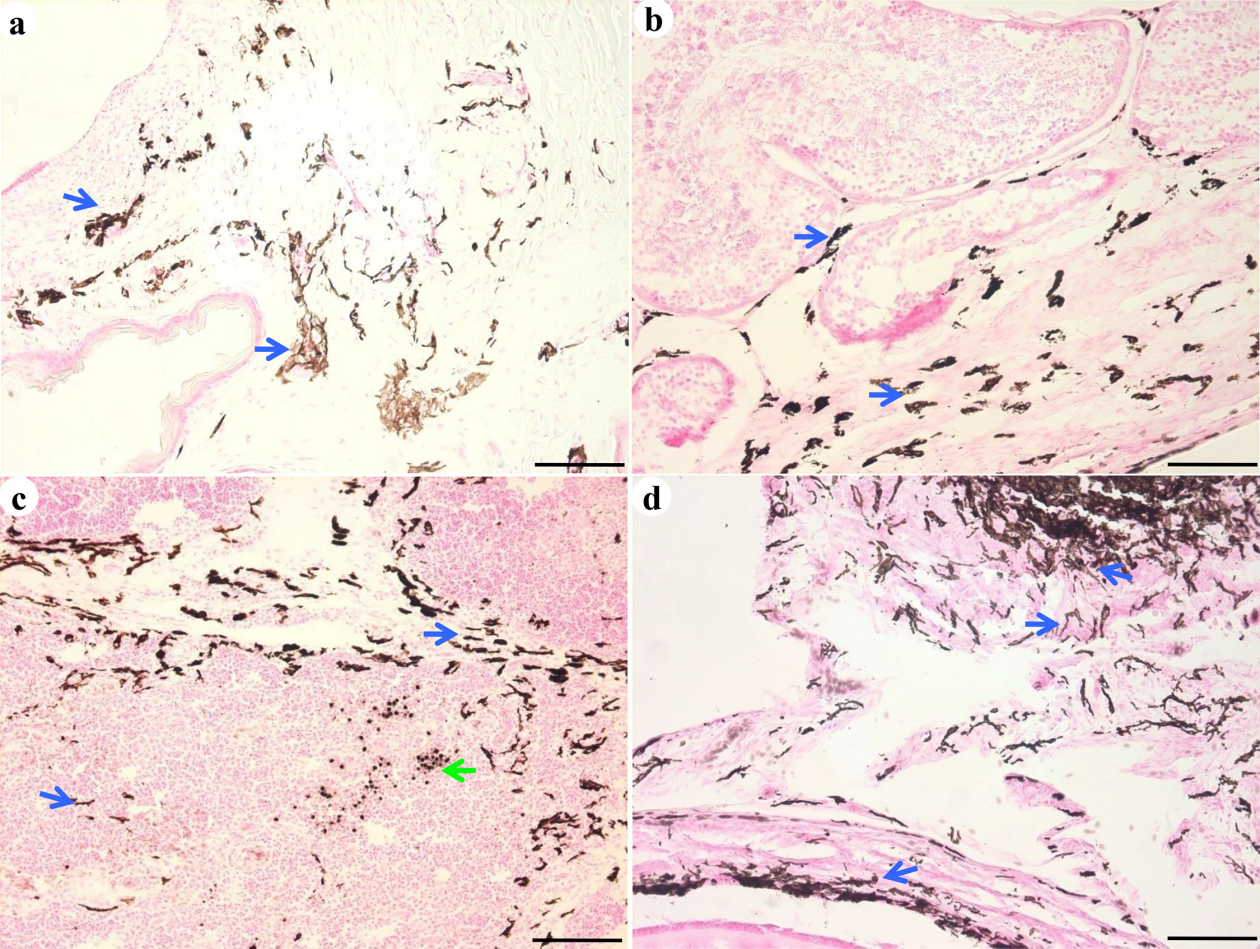
Melanocyte morphology. Numerous melanocytes (indicated by a blue arrow), with the appearance of dendritic cells and with round nuclei and abundant melanin, were observed in the skin (a), testis (b), thymus (c), and ovary (d). Macrophages in the thymus were stained by 3, 4-dihydroxy-l-phenylalanine (DOPA; c, green arrow). Scale bar = 100 μm.

After dissection and comparison with WLs, significant distribution of melanin was visible in multiple SF tissues by anatomy, including the skin, connective tissues (periosteum, mesentery, epicardium, meninx, and peritoneum), skeletal muscle (breast muscle and leg muscle), respiratory tract (trachea and lung), immune organs (spleen, thymus, and bursa of Fabricius), intestines, and reproductive organs (oviduct, ovary, vas deferens, and testis). Increased pigmentation was observed in the SFs with the growth of age from day 1 to week 23. Histological observations on the sections revealed large number of melanocytes in the skin, thymus, bursa of Fabricius, trachea, glandular stomach, ovary, and testis. Moderate amounts of melanocytes were present in the lungs, kidneys, spleen, oviducts, esophagus, muscular stomach, intestines, and leg muscles (Fig 2). A few melanocytes were detected in the breast muscle, heart muscle, vas deferens, and brain. Melanocytes were not detected in the liver, pancreas, pituitary gland, or adrenal gland. The morphology of the melanocytes was characteristic of dendritic cells, with round nuclei and abundant melanin in the cytoplasm (Fig 3, S1 Fig).

### Melanocyte localizationin the tissuesin SF

Melanocytes in the tissues were localized by observation of the sections. They were mainly present near blood vessels (S2 Fig), although specific distribution patterns were also observed in certain tissues. For example, melanocytes were mainly observed around the blood vessels of the lungs and the brain. But they were observed only in the outer membrane of the spleen, and they were present in the lymphoid nodule and trabecula of the thymus, and in the lymphoid nodule of the bursa of Fabricius. Numerous melanocytes were observed between the granular cells and the outer membrane of the ovary, and in the capsule and mesenchyme of the testis. Melanocytes were observed in the lamina propria of the mucosa in the glandular stomach and in the muscle layer and outer membrane of the intestines. In mice and humans, the primary locations of melanocytes are the epidermis of the skin. In contrast, in SF, melanocytes were only observed in the dermis.

**Fig 4:**
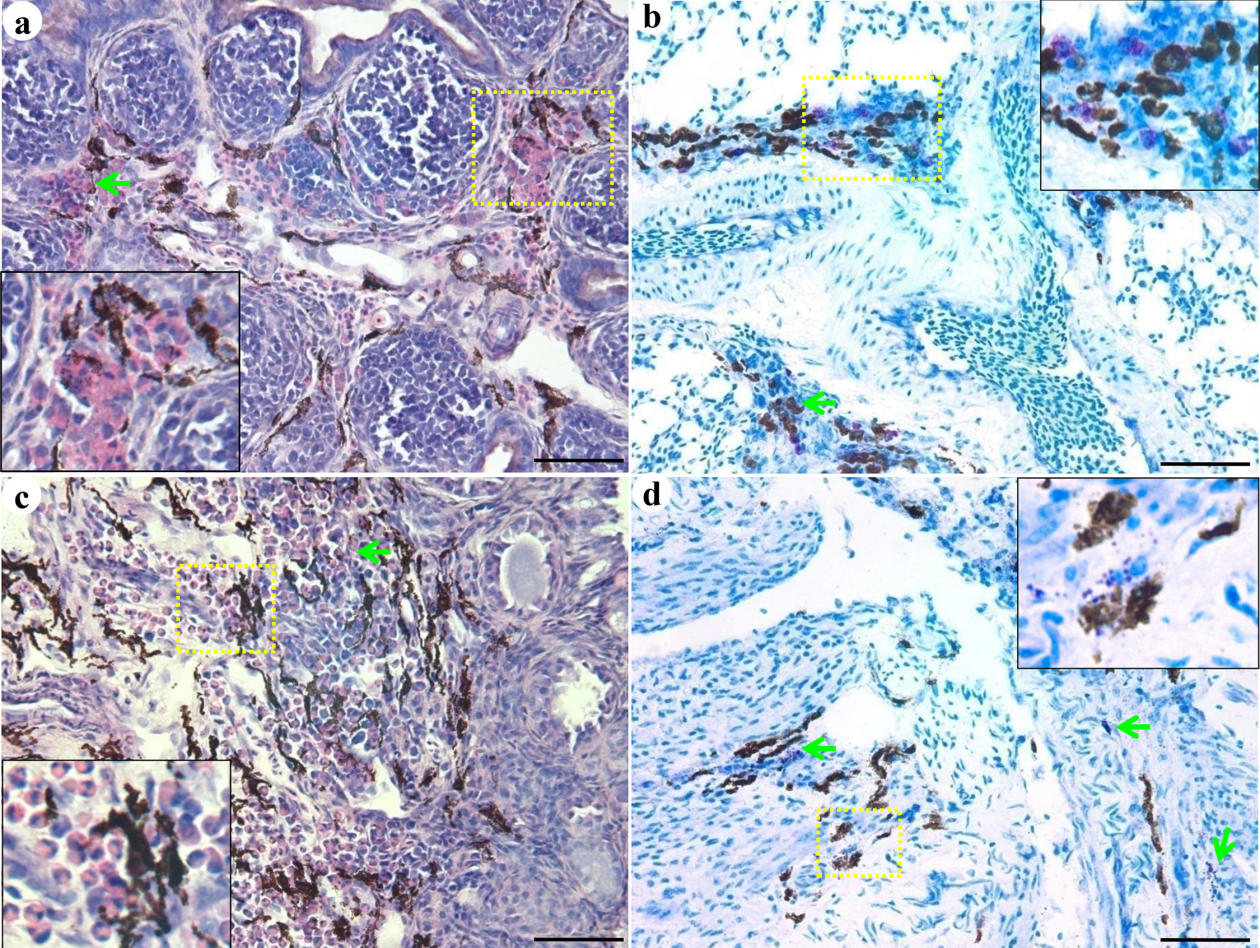
Tissue-dependent co-localization of melanocytes with heterophils and mast cells. Melanocytes co-localized with heterophils in the bursa of Fabricius (a) and ovaries (b). H&E stain. Melanocytes co-localized with mast cells in the lungs (c) and ovaries (d). Toluidine blue stain. Scale bar = 100 μm.

We next identified the cells that co-localized with the melanocytes. Toluidine-blue staining revealed numerous mast cells and melanocytes in the lungs (Fig 4B) and ovaries (Fig 4D), and the mast cells showed obvious degranulation in the ovaries. After H&E staining, numerous melanocytes and heterophils were observed in the bursa of Fabricius (Fig 4A) and ovaries (Fig 4C).

### Ultrastructural characteristics of the melanocytes

**Fig 5:**
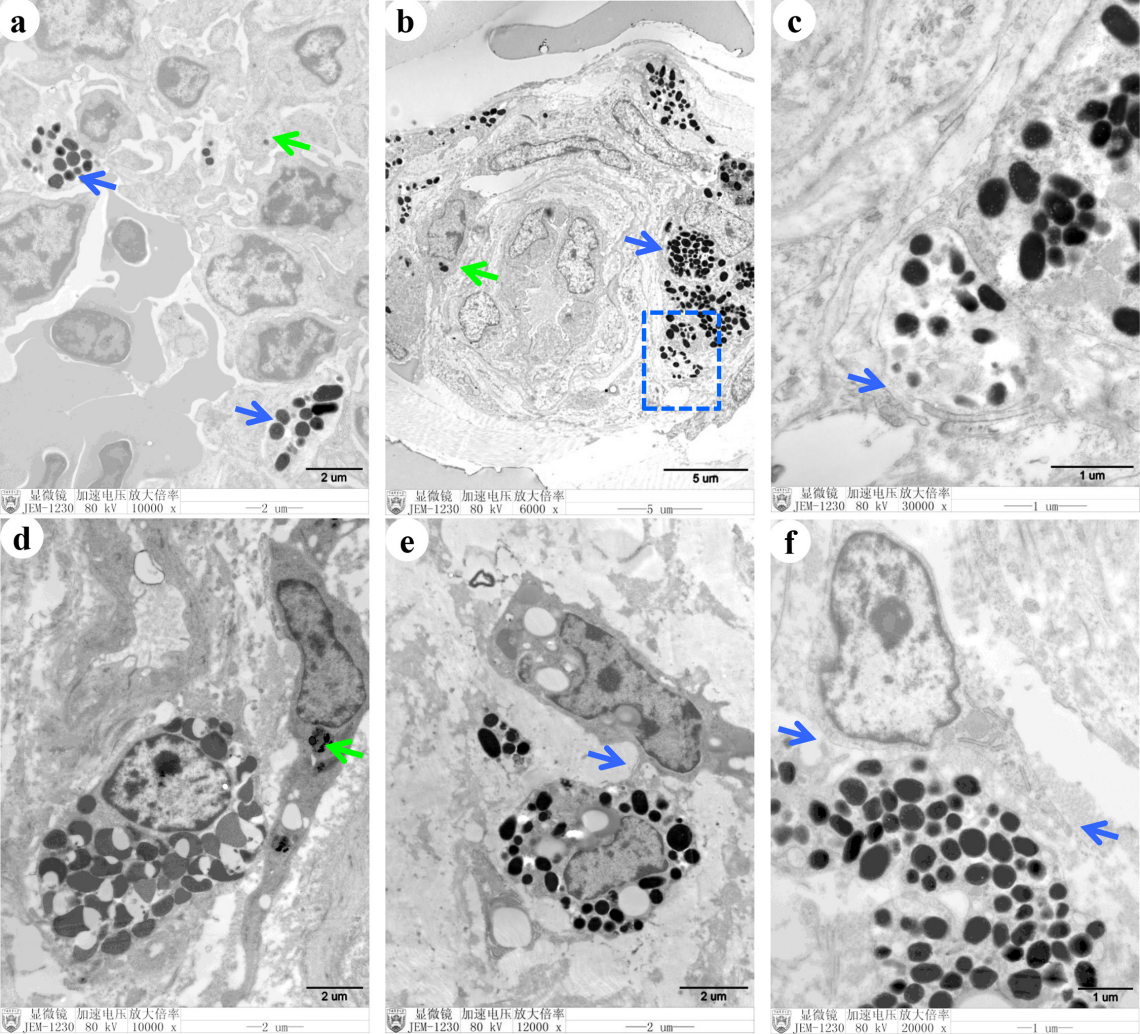
Tissue-specific ultrastructural characteristics of melanocytes and neighboring cells. Transmission electron microscopy was used to observe the melanocytes. Melanosomes were observed in the melanocytes (blue arrow) and the immune cells (green arrow) of the thymus (a). Melanosomes were present in other cells (green arrow) in the skin (b). Melanosomes were secreted by exocytosis from melanocytes in the skin (c). Melanosomes were observed in the interstitial cells (green arrow) in the ovaries (d). Melanocytes and neighboring cells were connected by dendrites in the ovaries (e). Long dendrites were detected in melanocytes and neighboring cells in the ovaries (f).

Transmission electron microscopy was used to examine the ultrastructural characteristics of melanocytes. Obvious melanosomes were observed in the melanocytes (Fig 5), and different stages of melanosomes were observed in the lungs and ovaries of 23-week-old SFs (S3 Fig). Numerous melanosomes were distributed among the tissue cells of the thymus (Fig 5A) and skin (Fig 5B). The melanosomes were secreted from melanocytes by exocytosis (Fig 5C). Moreover, melanocytes with cytoplasmic melanosomes contacted neighboring cells directly through dendrites (Fig 5E and 5F). Interestingly, the melanosomes were transferred and internalized between the cells, and the internalized melanosomes were attached to organelles, and not scattered throughout the cytoplasm (Fig 5A, 5B and 5D).

### Inhibition of the early development of the immune system

**Fig 6:**
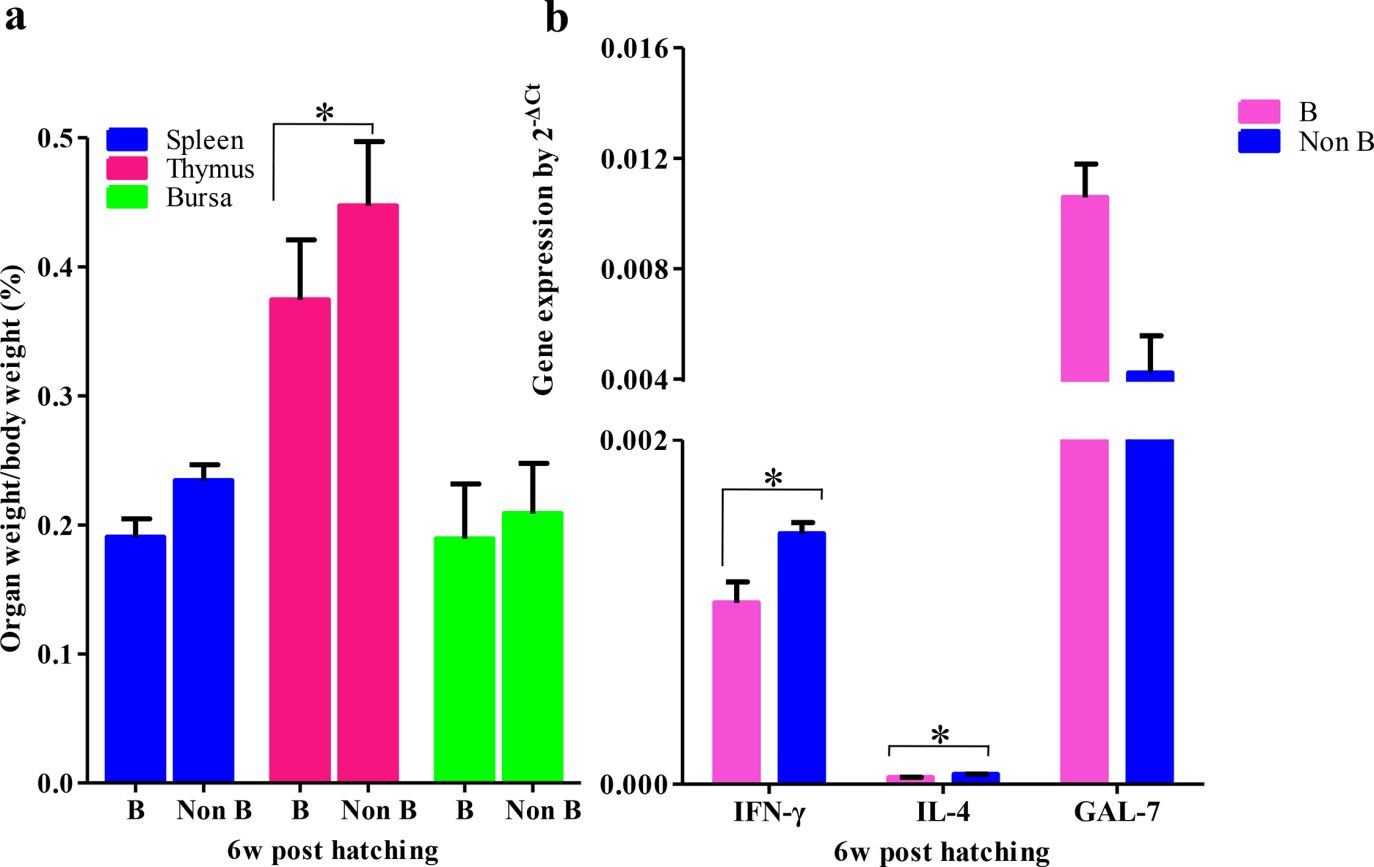
Organ indices and immune gene expression in the F2 population. (a) Lower indices of spleen, thymus, and bursa of Fabricius were detected in Black-boned chickens, with a significance difference (*P* < 0.05) in the thymus. (b) Lower expression levels of *IFN-γ* and *IL-4* were detected in Black-boned chickens, compared with those in non-Black-boned chickens (*P* < 0.05). Higher expression level of *GAL-7* was detected in Black-boned chickens, compared with that in non-Black-boned chickens, but the difference was not statistically significant. B, Black-boned chicken. Non B, non-Black-boned chicken.

Because atypical distribution of melanocytes was observed among the internal organs in the SFs, we determined the organ indices of the spleen, thymus, and bursa of Fabricius to ascertain whether melanocyte migration during embryogenesis influenced the development of the immune organs. A significant decrease in the indices of the spleen, thymus, and bursa of Fabricius were detected in the SFs before 6 weeks, compared with the WLs (Table 2). In order to eliminate the variation from genetic background, we also detected the indices of the spleen, thymus, and bursa of Fabricius in the Black-boned and non-Black-boned chickens in the F2 population and found lower indices of immune organs in the Black-boned chickens at week 6 (Fig 6). At 23 weeks of age, no significant difference of the indices of the spleen, thymus were detected, furthermore, higher index of bursa of Fabricius was detected in the SFs (Table 2).

**Table 2:** Organ indices of the spleen, thymus, and bursa of Fabricius of the SFs and WLs at different ages.

Breed	Age	Spleen	Thymus	Bursa of Fabricius
**SF male**	2 weeks	0.1180 ± 0.03	0.3808 ± 0.12	0.2801 ± 0.06**
**WL male**		0.1751 ± 0.04	0.3156 ± 0.05	0.4203 ± 0.04
**SF female**		0.1250 ± 0.01*	0.3976 ± 0.07	0.1720 ± 0.06**
**WL female**		0.2604 ± 0.10	0.2650 ± 0.00	0.4590 ± 0.02
**SF male**	3 weeks	0.1717 ± 0.07	0.3112 ± 0.12	0.2088 ± 0.15
**WL male**		0.3317 ± 0.08	0.2736 ± 0.07	0.2899 ± 0.12
**SF female**		0.1785 ± 0.04	0.3136 ± 0.04	0.2538 ± 0.02
**WL female**		0.2087 ± 0.03	0.2949 ± 0.04	0.1953 ± 0.02
**SF male**	4 weeks	0.2207 ± 0.02	0.3423 ± 0.05	0.1600 ± 0.02
**WL male**		0.2835 ± 0.07	0.3823 ± 0.05	0.1794 ± 0.01
**SF female**		0.1982 ± 0.02	0.3424 ± 0.13	0.1149 ± 0.01
**WL female**		0.3236 ± 0.06	0.2774 ± 0.06	0.1596 ± 0.02
**SF male**	6 weeks	0.1240 ± 0.03*	0.1963 ± 0.07	0.0890 ± 0.02
**WL male**		0.2126 ± 0.03	0.2767 ± 0.12	0.1120 ± 0.05
**SF female**		0.1779 ± 0.03	0.1744 ± 0.05**	0.0880 ± 0.02
**WL female**		0.1999 ± 0.05	0.2569 ± 0.05	0.1671 ± 0.09
**SF male**	10 weeks	0.1551 ± 0.02	0.2826 ± 0.07	0.1073 ± 0.03
**WL male**		0.2388 ± 0.08	0.2251 ± 0.04	0.2629 ± 0.06
**SF female**		0.2023 ± 0.06	0.2283 ± 0.06	0.0861 ± 0.02
**WL female**		0.2483 ± 0.06	0.2403 ± 0.08	0.1824 ± 0.09
**SF male**	23 weeks	0.1114 ± 0.02	0.1068 ± 0.02	0.0186 ± 0.00**
**WL male**		0.1420 ± 0.03	0.0976 ± 0.02	0.0023 ± 0.00
**SF female**		0.0593 ± 0.00	0.0316 ± 0.00	0.0079 ± 0.00**
**WL female**		0.1809 ± 0.06	0.0349 ± 0.00	0.0002 ± 0.00

Note: **P <0.01 and *P <0.05 indicates statistically significant differences between SF and WL in the same gender.

**Fig 7:**
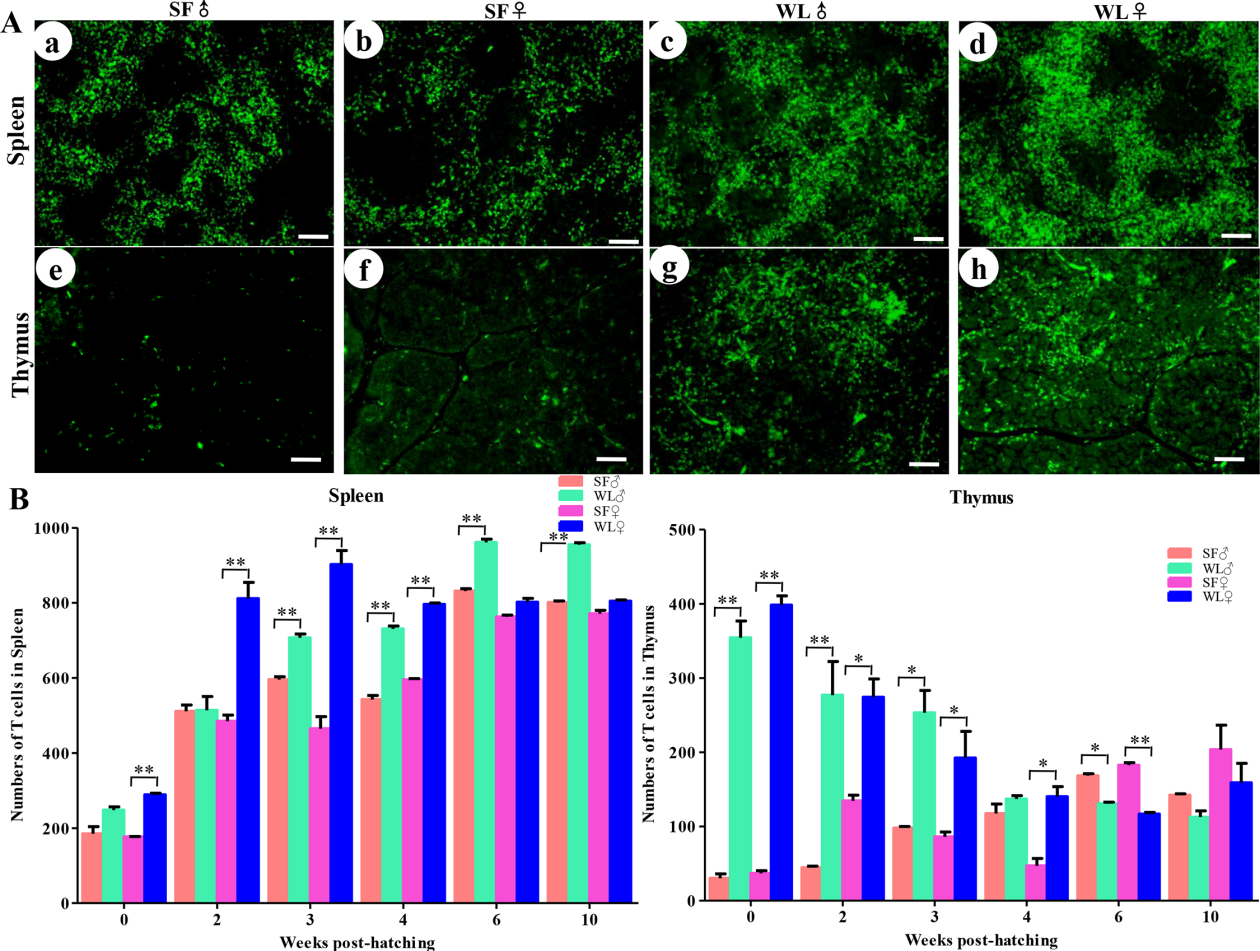
CD3^+^ cells in the spleen and thymus of SF and WL. (A) CD3^+^ cells were observed in the spleen (a, b) and thymus (e, f) of SF and in the spleen (c, d) and thymus (g, h) of WL. Scale bar = 100 μm. (B) There were significant differences during early development between the numbers of CD3^+^ cells in the spleen and thymus of SF and WL. *P < 0.05, **P < 0.01.

**Fig 8:**
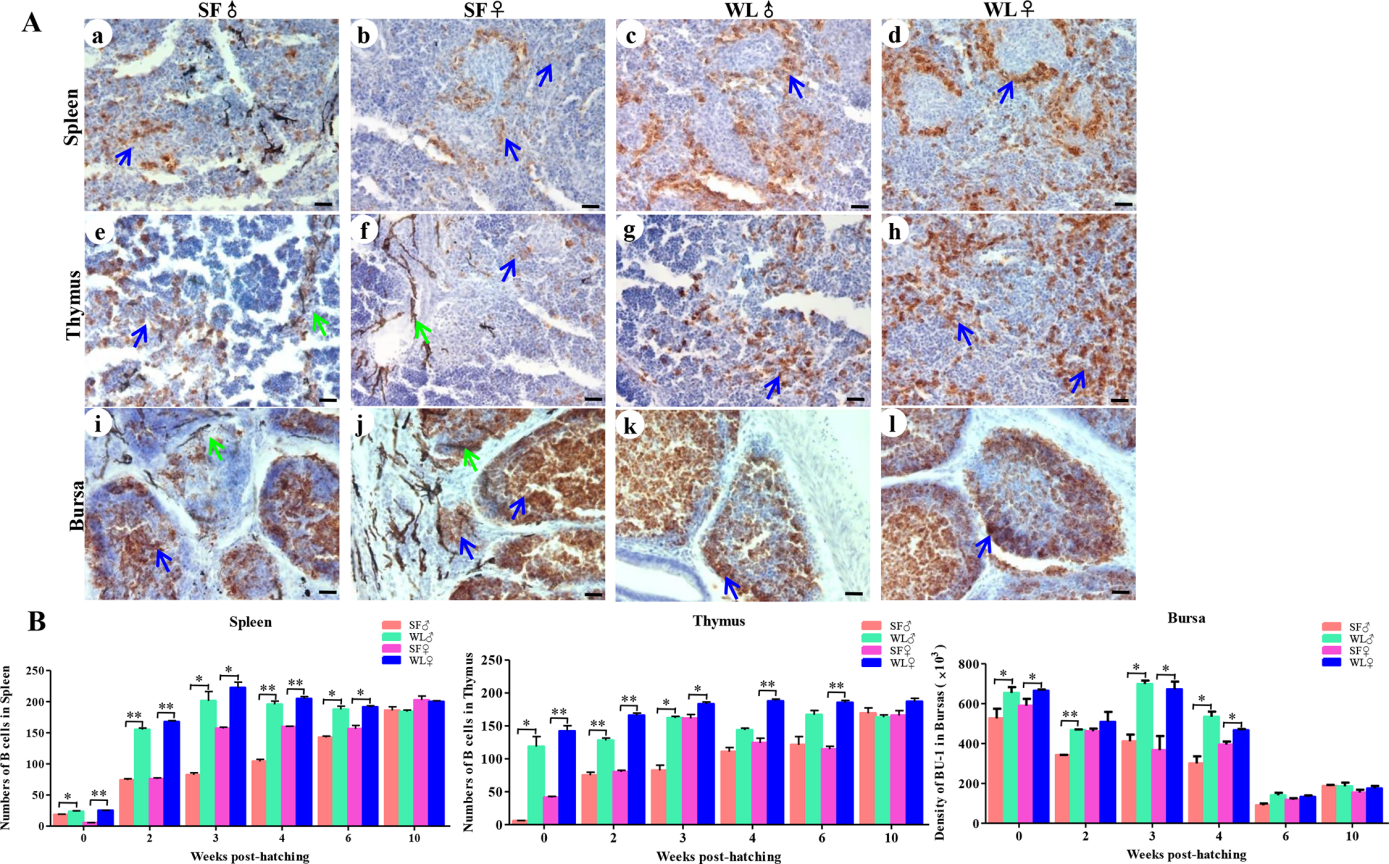
Bu-1^+^ cells in the spleen, thymus, and bursa of Fabricius of SF and WL. (A) Bu-1^+^ cells were observed in the spleen (a, b), thymus (e, f), and bursa of Fabricius (i, j) of SF and in the spleen (c, d), thymus (g, h), and bursa of Fabricius (k, l) of WL. Scale bar = 100 μm. (B) There were significant differences in the numbers of Bu-1^+^ cells in the spleen, thymus, and bursa of Fabricius of SF, compared with those in WL, during early development. *P < 0.05, **P<0.01.

We further detected the numbers of immune cells in the spleen, thymus, and bursa of Fabricius. The number of CD3^+^ cells in the spleen and thymus was significantly lower in the SFs than that in the WLs, during the early developmental stages (Fig 7A), and the differences were significant between the two breeds from day 1 to 6 weeks of age (*P* < 0.01). The number of Bu-1^+^ cells in the spleen, thymus, and bursa of Fabricius was also lower in the early stage of the SFs (Fig 8A), and the differences were significant from day 1 to week 6 between SFs and WLs.

**Fig 9:**
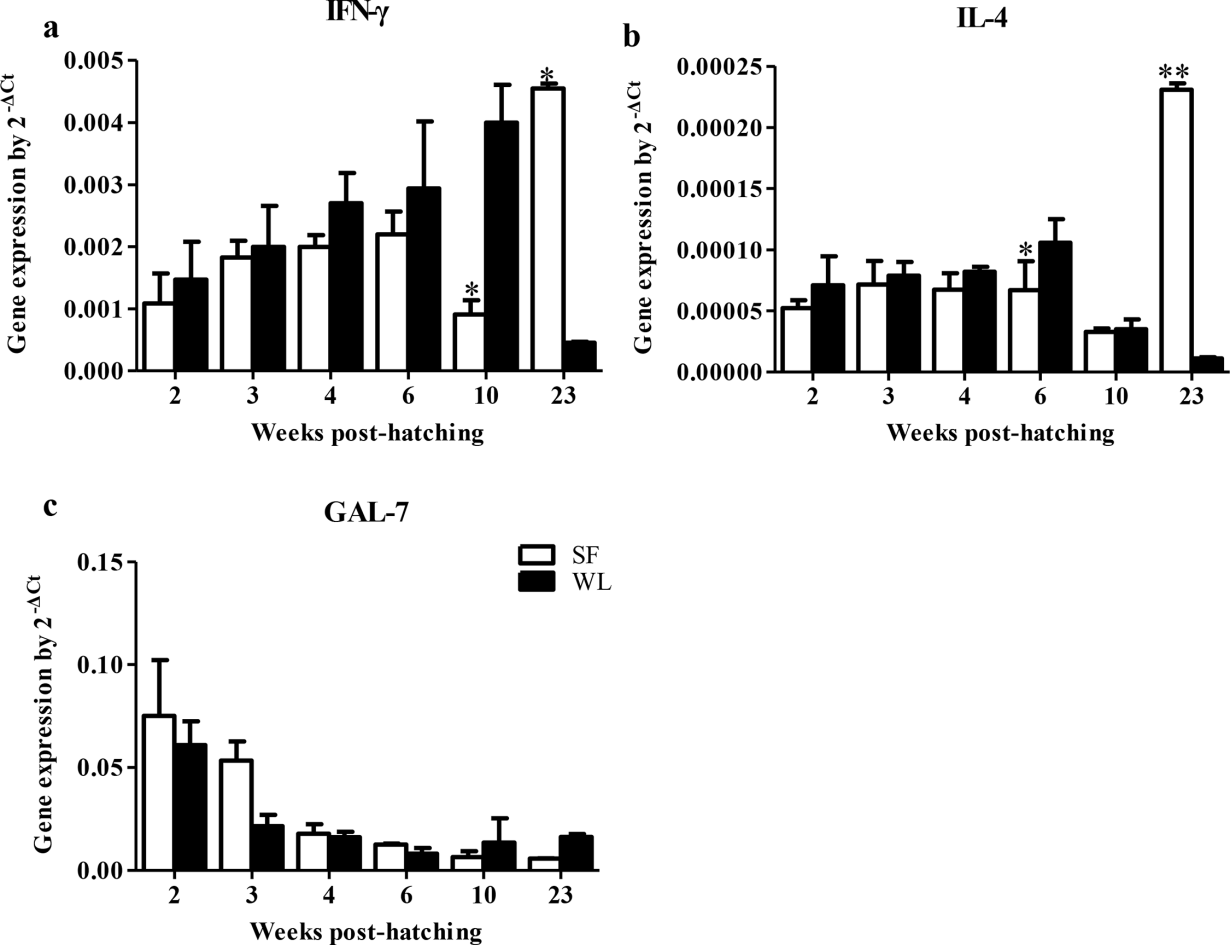
Expression of *IFN-γ*, *IL-4* and *GAL-7* in the spleen of SF and WL. Lower expression levels of *IFN-γ* (a) and *IL-4* (b) were detected in SF at 10 and 6 weeks of age, respectively, but higher expression levels were detected in SF at 23 weeks of age. High expression level of *GAL*-7 was detected in SF before 6 weeks of age, but low expression levels were detected at 10 and 23 weeks of age. (c). *P < 0.05, **P < 0.01.

In order to identify the mechanisms underlying immune inhibition in the SFs, immune factors *IFN-γ*, *IL-4* and *GAL-7* expression in the spleen were quantified. As shown in Fig 9, low expression levels of *IFN-γ* and *IL-4* were detected from 2 to 10 weeks old in SFs, and significant differences were detected at 6 and 10 weeks of age (*P* < 0.05). No significant difference of *GAL-7* expression level was detected between SFs and WLs. Similar differences in the expression levels of *IFN-γ*, *IL-4* and *GAL-7* were detected between the Black-boned and non-Black-boned chickens from the hybrid F2 population (Fig 6). The expression levels of *IFN-γ* and *IL-4* were significantly lower in the Black-boned chickens than that in the non-Black-boned chickens (*P* < 0.05).

After immunizing the chickens with H5N1 Re4 and IBDV vaccines, antibody titers were estimated in order to investigate the differences in antibody production between the SFs and WLs. As shown in Fig 1, lower levels of antibodies were detected in the serum of SFs, and significant differences in the antibody levels against IBDV were observed in SFs at 6 and 10 weeks of age (*P* < 0.05).

### Minor apoptotic effect in SF

**Fig 10:**
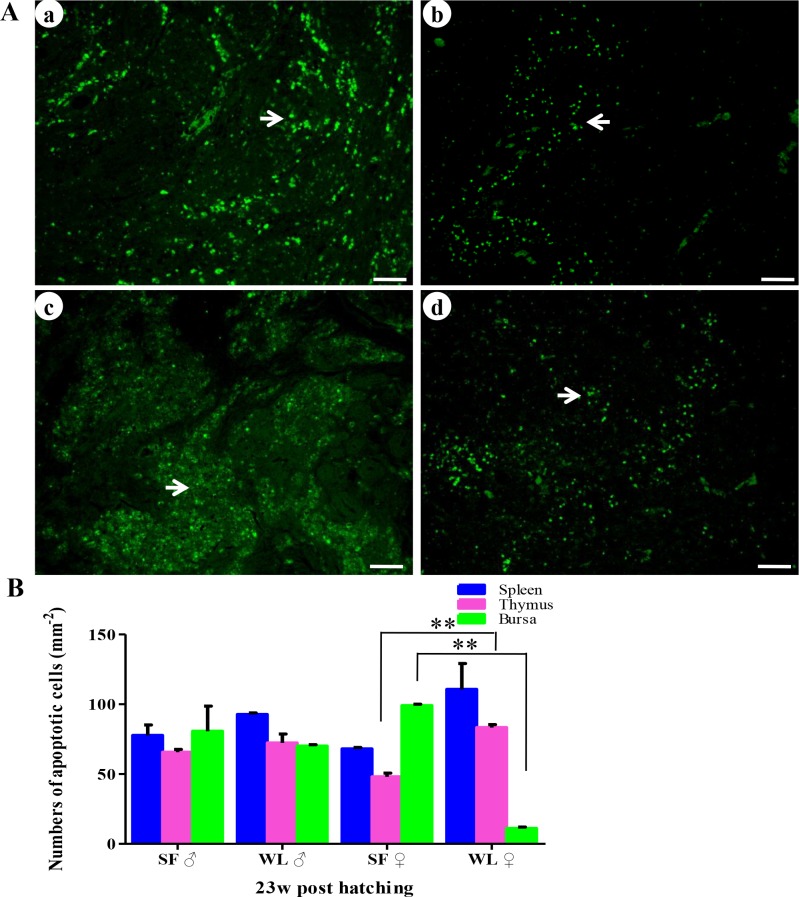
TUNEL analysis of apoptosis in SF and WL. (A) Apoptotic cells were observed in the bursa of Fabricius (a) and thymus (b) of SF, and the bursa of Fabricius (c) and thymus (d) of WL. Scale bar = 100 μm. (B) There were significant differences between the numbers of apoptotic cells in the spleen, thymus, and bursa of Fabricius in SF, compared with those in WL. **P<0.01.

After sexual maturation, the organ indices of the spleen, thymus did not show significant difference between SFs and WLs, while the index of the bursa of Fabricius was even higher in SFs than in WLs (Table 2), indicating the rate of atrophy of the immune organs was slower in the SFs than in the WLs. We analyzed apoptosis in the spleen, thymus, and bursa of Fabricius in the chickens at 23 weeks of age. The TUNEL assays detected more apoptotic cells in the spleen of WLs, compared with the SFs, but the difference was not statistically significant (Fig 10B). Fewer apoptotic cells were observed in the thymus of the SFs, compared with the WLs (Fig 10A), and there was a significant difference between the females of the two breeds (*P* < 0.01). In contrast, more apoptotic cells were observed in the bursa of Fabricius in the SFs (Fig 10A), and significant difference was detected between the females of the two breeds (*P* < 0.01).

**Fig 11:**
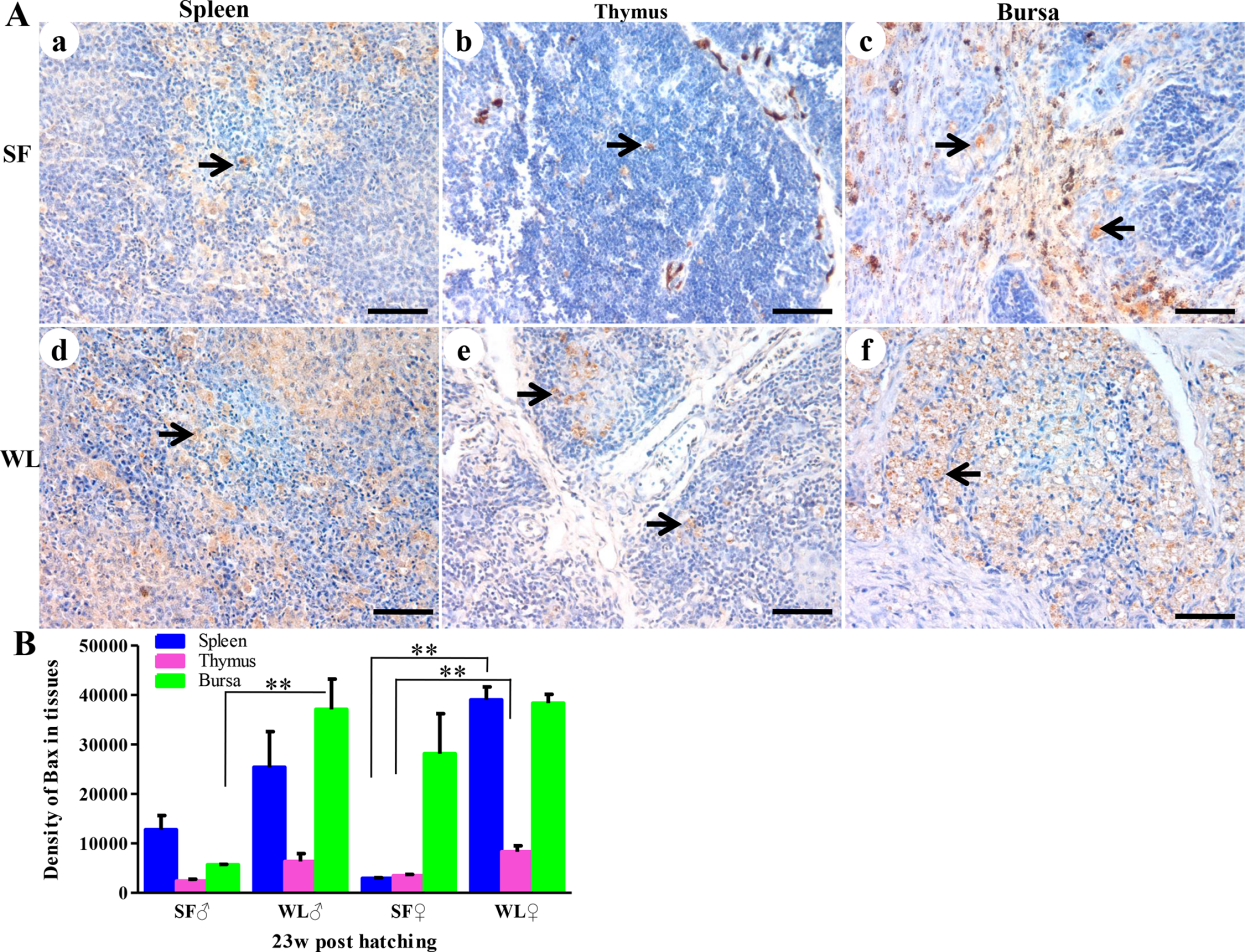
Immunohistochemical analysis of Bax expression. (A) Bax expression (brown) was detected in the spleen (a), thymus (b), and bursa of Fabricius (c) and of SF and the spleen (d), thymus (e), and bursa of Fabricius (f) of WL. Scale bar = 100 μm. (B) There was a significant difference in the numbers of Bax^+^ cells in the spleen, thymus, and bursa of Fabricius of SF, compared with those of WL. **P<0.01.

The expression levels of Bax and Bcl-2 proteins were determined to identify the mechanism of apoptosis in the spleen, thymus, and bursa of Fabricius of the SFs and WLs at 23 weeks of age. After immunohistochemical analysis, no significant differences in anti-apoptotic Bcl-2 protein expression were detected between the SFs and WLs. However, higher levels of pro-apoptotic Bax proteins were detected in the spleen, thymus, and bursa of Fabricius of WLs (Fig 11A). There were significant differences in Bax expression in the bursa of Fabricius between the male chickens of SFs and WLs (*P* <0.01), and obvious differences in the spleen and thymus between the female chickens of the two breeds (*P* < 0.01; Fig 11B).

## Discussion

Melanoblasts originate from neural crest cells, migrate through the mesenchyme of the developed embryo, and give rise to melanocytes [22]. Unlike the melanocytes in other vertebrates, which are confined to the integument by dorsal migration, melanocytes can reach the inner organs of SF by ventral migration [23].Several previous studies have revealed pigments in the skin, heart, kidney, liver, gizzard, cecum, trachea, gonads, and periosteum of SF [24]. In this study, we found that in addition to the above-reported organs, melanocytes are present in almost all the inner organs, including the spleen, bursa of Fabricius, thymus, lung, brain etc. Furthermore, melanocytes were not observed in the liver, pancreas, hypothalamus, pituitary gland, and adrenal gland in our study. Detection of melanin in the liver is inconsistent with the results of previous reports, which may be attributed to the fact that melanocytes are located within the capsule, and not in the parenchyma. Interestingly, melanocytes in different tissues were mainly located near blood vessels. This might be associated with the migration pathway of the neural crest cells. Moreover, melanin synthesis results from the combination of the α-melanocyte stimulating hormone (α-MSH) and the adrenocorticotrophic hormone (ACTH) with the Mc1R on the membranes of melanocytes [25]. Therefore, we hypothesize that melanin synthesis and melanocyte function may be regulated by hormones present in the blood.

In a previous study, different amounts of melanin were detected in the inner organs of SF [24]. Consistent with this finding, we observed that the number of melanocytes varied among the inner organs. In addition, the number of melanocytes in an organ changed during the development of the chicken (S4 Fig). Additionally, at the early stage of development, melanin was mainly confined in the melanocytes, but at 23 weeks of age, numerous melanin pigments were observed in the bursa of Fabricius (lamina propria) and ovaries (corpus luteum). These results indicated that the melanocytes proliferated after hatching, and that the function of melanocytes might be mediated via secreted melanin. Melanocytes mainly populate the epidermis and hair follicles in mammals to shield the organism from sunlight and maintain hair color [26–28]. However, in SFs, skin melanocytes are present only in the dermis and not in the follicles. Therefore, melanocytes do not contribute to feather color of SF (which is white). As reported previously in the skin of SF embryos [6], melanocytes with round nuclei and long dendrites were observed in the inner organs of the SFs at different ages in our study (Fig 2, S1 Fig). Therefore, no significant differences were observed in melanocyte phenotypes between the skin and inner organs. Ultrastructural observation revealed that melanosomes were not confined to melanocytes. Melanosomes were also observed in keratinocytes, lymphocytes in thymus and bursa of Fabricius, and tissue cells in ovary, which were attached to cytoplasmic organelles such as mitochondria. Ortolani-Machado reported that macrophages present in the dermis of SF embryos contain melanin granules in their cytoplasm [6]. Using atomic force microscopy, Ma and co-workers demonstrated that melanosomes transferred to keratinocytes by promoting filopodia delivery and shedding spheroid granules [29]. In this study, contact between melanocytes and tissues cells by filopodia, and between melanocytes and granules by exocytosis were observed in the skin and inner organs by transmission electron microscopy. These results suggest that melanocytes communicate with other cells via filopodia to complete melanosome delivery. In a previous study, in the dermis of SF embryos, melanosomes at different stages of maturation were observed in melanocytes during development [7]. In this study, melanosomes at different maturation stages were observed in the inner organs of 23-week-old SFs (S3 Fig). This finding suggests that melanin synthesis in the melanocytes continues after hatching. Moreover, one may conclude that the melanin found in the inner organs of SF is mainly eumelanin, generated by melanosome morphogenesis. This hypothesis is in accordance with the findings of previous studies [30, 31]. Next, we investigated the roles of melanocytes in the organs of SF during development. The melanocytes in different tissues were distributed mainly around the blood vessels, especially in the brain, lung, and testis, which constitute the blood-brain, blood-air, and blood-testis barriers, respectively (S2 Fig). In humans, perivascular-resident macrophage-like melanocytes in the inner ear play essential roles in maintaining the integrity of the interstitial fluid-blood barrier [13]. Both melanocytes and macrophages were stained by DOPA, suggesting that these cell types express tyrosinases and they might have common roles. A few previous studies have demonstrated that melanocytes in zebra fish migrate into the wound caused by exogenous beading implantation and encapsulate the graft after infiltration by neutrophils and macrophages [14, 32]. Moreover, in the present study, melanocytes were observed mainly near blood vessels, as were mast cells. Although a direct connection between mast cells and melanocytes was not detected in our analysis, co-localization of degranulated mast cells and melanocytes was observed in our study. Mast cells contribute to innate immunity by secreting inflammatory cytokines, and they play important roles in the pathogenesis of certain diseases [33–36]. Our present studies on SF suggest that melanocytes may participate in the immune response. Recently, additional evidence has suggested that, in addition to the skin, melanocyte-related cells are present in the ear and heart, where they play crucial roles in the process of hearing and heart disease in humans [13, 28]. Elucidating the functions of melanocytes in SFs might provide insights into the roles of melanocytes in humans and other mammals.

During the development of ovarian follicles in the SFs, melanocytes were not observed in primordial and primary follicles, but they were present in secondary and mature follicles, as well as in the corpus luteum (S5 Fig). This result suggests that melanocytes may be involved in the regulation of sex hormone secretion, and it may inhibit the development of ovarian follicles. Consistent with this hypothesis, lower egg production was observed in the SFs compared to other breeds in our farm (data not shown). Liu reported that the peak laying rate of SF is lower than that of other breeds (48.81%, *P* < 0.01) [37].

Finally, as central components of the immune system of chickens, the thymus and bursa of Fabricius provide sites for the development of T and B lymphocyte. However, significantly fewer immune cells were detected in the SFs, compared with the WLs. IL-4, which is produced mainly by T cells, plays crucial roles in the immune regulation of B cells, mast cells, monocytes, and hematopoietic cells. IFN-γ, which is produced mainly by T cells and natural killer cells, has immunomodulatory effects on antigen presentation cells, macrophages, natural killer cells, and B cells. In our study, lower expression levels of *IFN-γ* and *IL-4* were detected in the spleen of SF, compared with WL. However, the expression level of *GAL-7*, an innate defensin, was relatively high in SF, suggesting that the observed expression levels may be a compensatory effect of the inhibition of adaptive immunity. Moreover, after immunization, lower antibody levels were detected in the serum of SF, indicating that the B cell response is weaker in SF than that in WL. Our results suggest that melanocyte migration is involved in the proliferation and differentiation of immune cells in immune organs, and in the regulation of genes involved in the immune system in the spleen. Additionally, similar results were obtained for immune organ indices and immune gene expressions in the F2 population, which further supports the hypothesis that inhibition of immune organ development is substantially related to the atypical melanocyte migration, rather than variations among different breeds. Our previous microarray study revealed that genes involved in the innate immune response are expressed at higher levels compared to those involved in the adoptive immune response during migration of melanocytes in the embryo on days 3, 3.5, 4, and 4.5 [19]. We concluded that melanocyte migration negatively regulates immune development in SF embryos, and that the influence continued at the early stage development. In addition, previous study found that corticotropin releasing hormone and POMC derived peptides had immunosuppressive properties such as α-MSH and ACTH which could stimulate cortisol production [38]. Moreover, as is reported that the melanin precursors L-tyrosine and L-dihydroxyphenylalanine may directly modulate the other cells’ function by binding the corresponding receptors or indirectly affect the other cells’ function by melanosomes delivery [39–41]. In our report, melanocytes proliferation and melanin synthesis after hatching were observed, which might result in more corticotropin releasing hormone and POMC derived peptides production. Moreover, that melanocytes mainly located near blood vessels facilitate these peptides releasing in the blood circulation to modulate immune cells development. Our results demonstrate that hyperpigmentation in SF have resulted in weakened immune development, as evidenced by the lower survival rate observed during breeding in the animal farm at the China Agricultural University (data not shown). Lower survival rates are also consistent with Liu’s study, survival rates of 78.28% at 6 weeks of age (*P* < 0.01) and 64.38% at 10 weeks of age (*P* < 0.01) were recorded [38]. Followed up studies on investigating the pleiotropic functions of melanin precursors could help us to better understand the precise mechanism of melanocytes’ role in modulating the immune organs development.

In addition, fewer apoptotic cells were observed in the spleen and thymus of SF compared with WL, and more apoptotic cells were present in the bursa of Fabricius, due to severe fibrosis in the bursa of Fabricius in WL. This result indicates that weak apoptosis delayed the degeneration of thymus and bursa of Fabricius in SF. Moreover, lower Bax expression were observed in the immune organs of SF, suggesting that down regulation of pro-apoptotic Bax expression may be related to the weaker apoptosis [42]. Furthermore, at 23 weeks of age, higher expression levels of *IFN-γ* and *IL-4* were detected in the spleen of SF. This might be correlated to the delayed degeneration of thymus and bursa of Fabricius. Additionally, different from melanocyte proliferation and melanin synthesis at the early stage of development, melanin has beneficial effects on the immune response and other biological functions during later stages. The ultrastructural examinations revealed melanosomes bound to the mitochondrial membrane. This finding corroborates our hypothesis that melanosomes regulate the Bax protein to inhibit apoptotic pathways, resulting in weaker apoptosis in SF. Further studies are required to confirm this conclusion. In addition, when the bursa of Fabricius degenerated at 23 weeks of age, lymphoid nodes containing Bu-1^+^ cells were detected in the spleen and intestines of SFs and WLs (S6 Fig). These cells might continue to play immune roles after bursa of Fabricius degeneration.

In humans, melanogenesis is a pathogenic factor in melanoma progression, and it results in an immunosuppressive environment [43]. Moreover, melanomas are caused by malignant transformation of melanocytes and inappropriate synthesis of melanin [44–46]. In this study, inhibition of immune development and wide distribution of melanocytes among internal organs were observed in SF. Moreover, higher mortalities of SF before 10 weeks old are observed compared with other breeds in the previous study [37] and in our farm during the breeding. These results suggest that similar mechanisms of immune inhibition underlie melanoma pathogenesis in humans and hyperpigmentation in SFs. Therefore, understanding the mechanisms underlying hyperpigmentation and melanocyte function in SF might provide insights into melanoma morphogenesis in humans. Collectively, the results of the present study revealed that melanocytes are widely distributed in the internal organs of SF and that melanocyte proliferation and melanin synthesis continue after hatching. Our findings indicate that hyperpigmentation in SF inhibits immune organ development at early stages, and the presence of melanin in the immune organs delays degeneration. Our present findings shed new light on the role of melanocytes in hyperpigmented SFs. The molecular mechanism of hyperpigmentation on regulation of tissue cells development needs further investigation.

## Supporting Information

S1 Fig Melanocyte characteristics in the thymus and ovary. The melanocytes were characterized by round nucleus, abundant cytoplasmic melanin, and long dendrites in the membrane. (a) Thymus, H&E stain. (b) Ovary, DOPA stain. Scale bar = 100μm.
(TIF)

S2 Fig Melanocytes are located near blood vessels. (a) Brain. (b) Heart muscle. (c) Lung. (d) Testis. Melanocyte (green arrow); Blood vessel (blue arrow). Scale bar = 100μm.
(TIF)

S3 Fig Melanosomes at different stages of maturity in melanocytes. In 23-week-old SFs, melanosomes (blue arrow) at different stages of maturity (shown as rounds or ovals) were observed. (a) Lung. (b–f) Ovary.
(TIF)

S4 Fig Changes in melanocyte phenotype in the thymus, bursa of Fabricius, testis, and ovary of SFs at 3, 6, and 23 weeks of age. After hatching, the number of melanocytes increased, but no significant changes were observed after maturity. Scale bar = 100 μm.
(TIF)

S5 Fig Melanocytes in follicles during development. Melanocytes were observed in the secondary and mature follicles and in the corpus luteum, but not in primordial and primary follicles. Scale bar = 100 μm.
(TIF)

S6 Fig Bu-1^+^ cells in the spleen and cecum. Lymphoid nodules with Bu-1^+^ cells in the spleen (a) and cecum (b). Scale bar = 100 μm.
(TIF)
