# Supplementary figures and images for: Hyperpigmentation Results in Aberrant Immune Development in Silky Fowl (Gallus gallus domesticus Brisson)
Source: PLoS One. 2015 Jun 5;10(6):e0125686. doi: 10.1371/journal.pone.0125686 (PMC4457905; doi:10.1371/journal.pone.0125686)

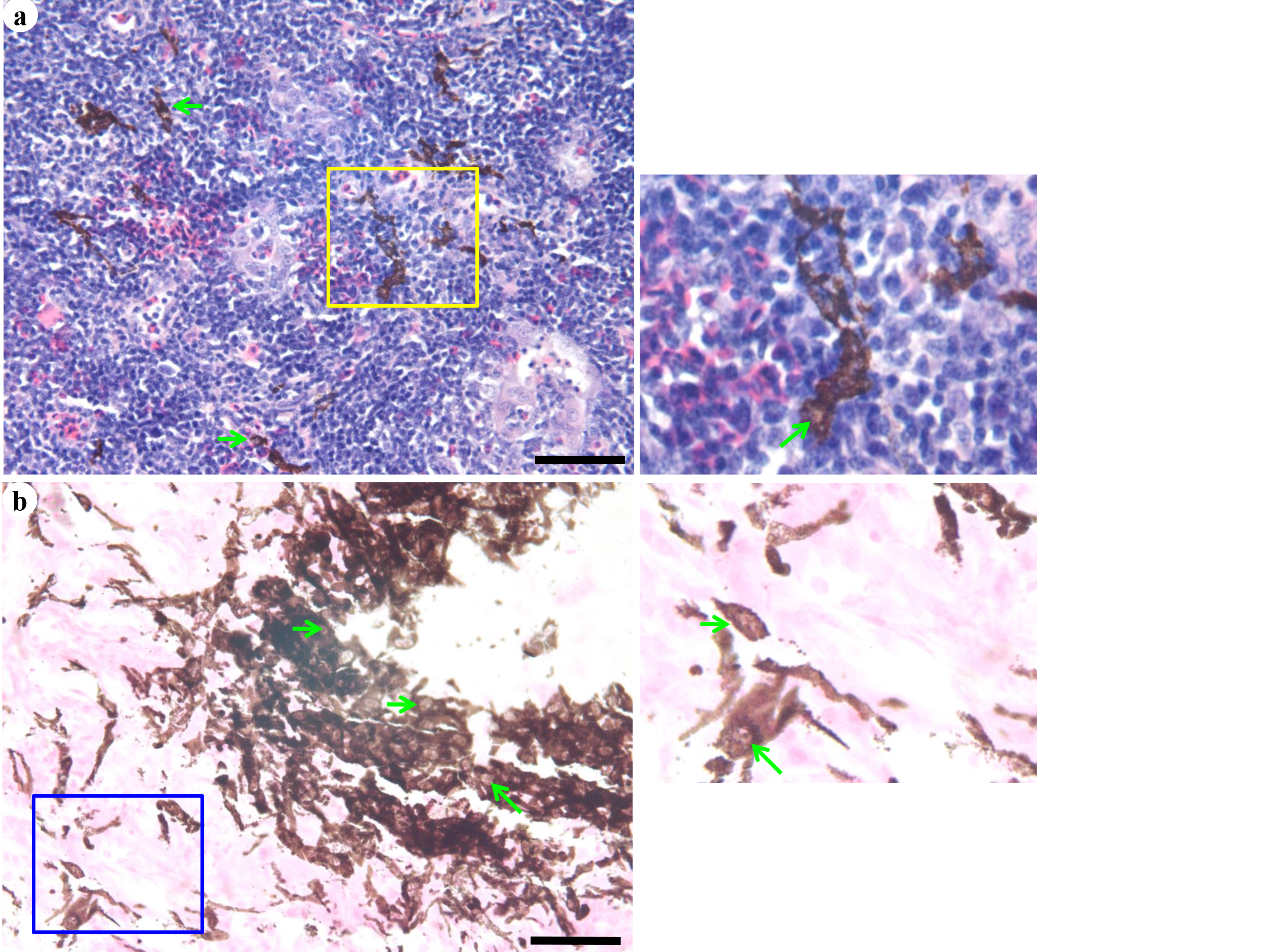

Supplement: S1 Fig — The melanocytes were characterized by round nucleus, abundant cytoplasmic melanin, and long dendrites in the membrane. (a) Thymus, H&E stain. (b) Ovary, DOPA stain. Scale bar = 100μm. (TIF) [file pone.0125686.s001.tif]

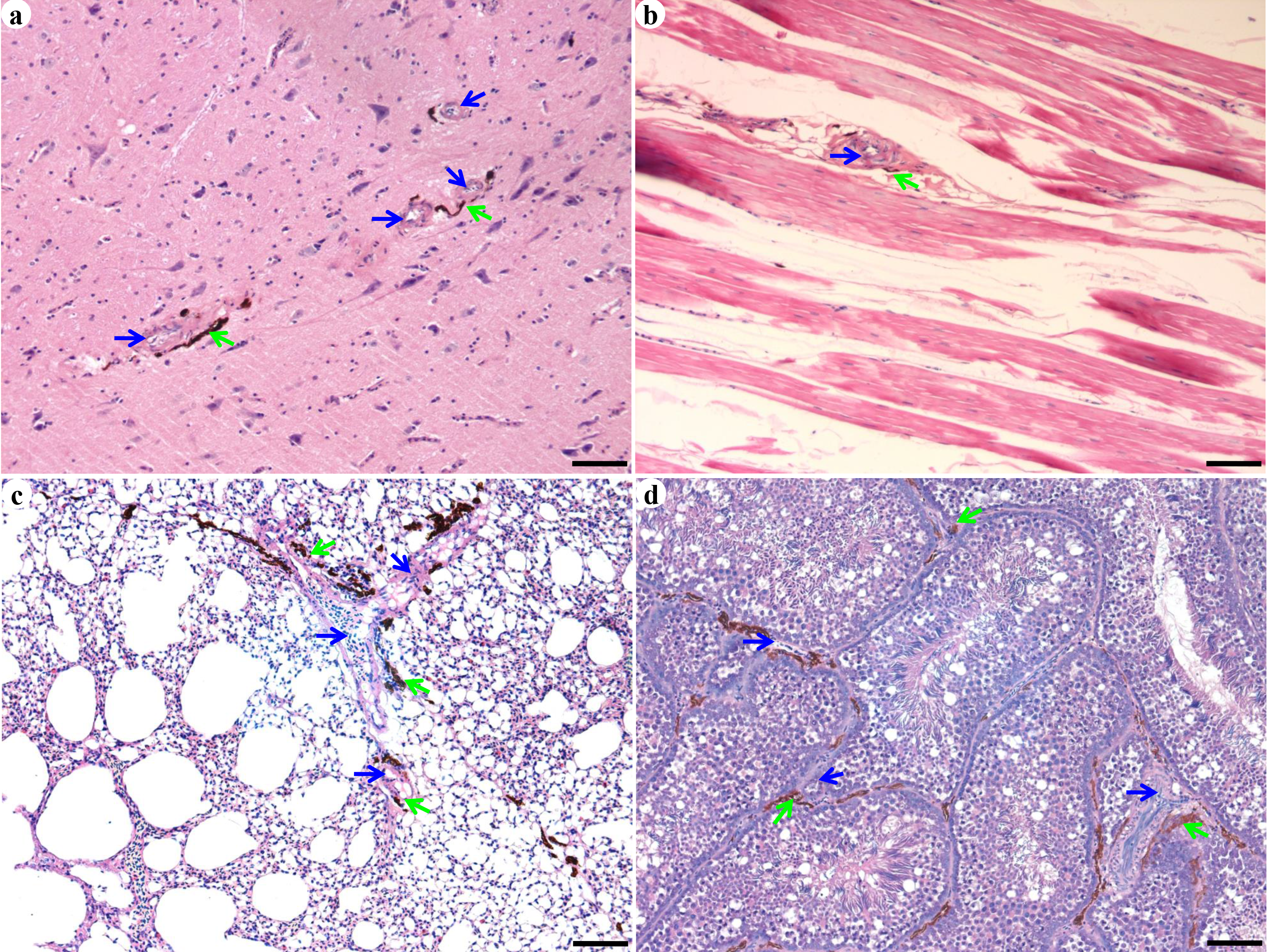

Supplement: S2 Fig — (a) Brain. (b) Heart muscle. (c) Lung. (d) Testis. Melanocyte (green arrow); Blood vessel (blue arrow). Scale bar = 100μm. (TIF) [file pone.0125686.s002.tif]

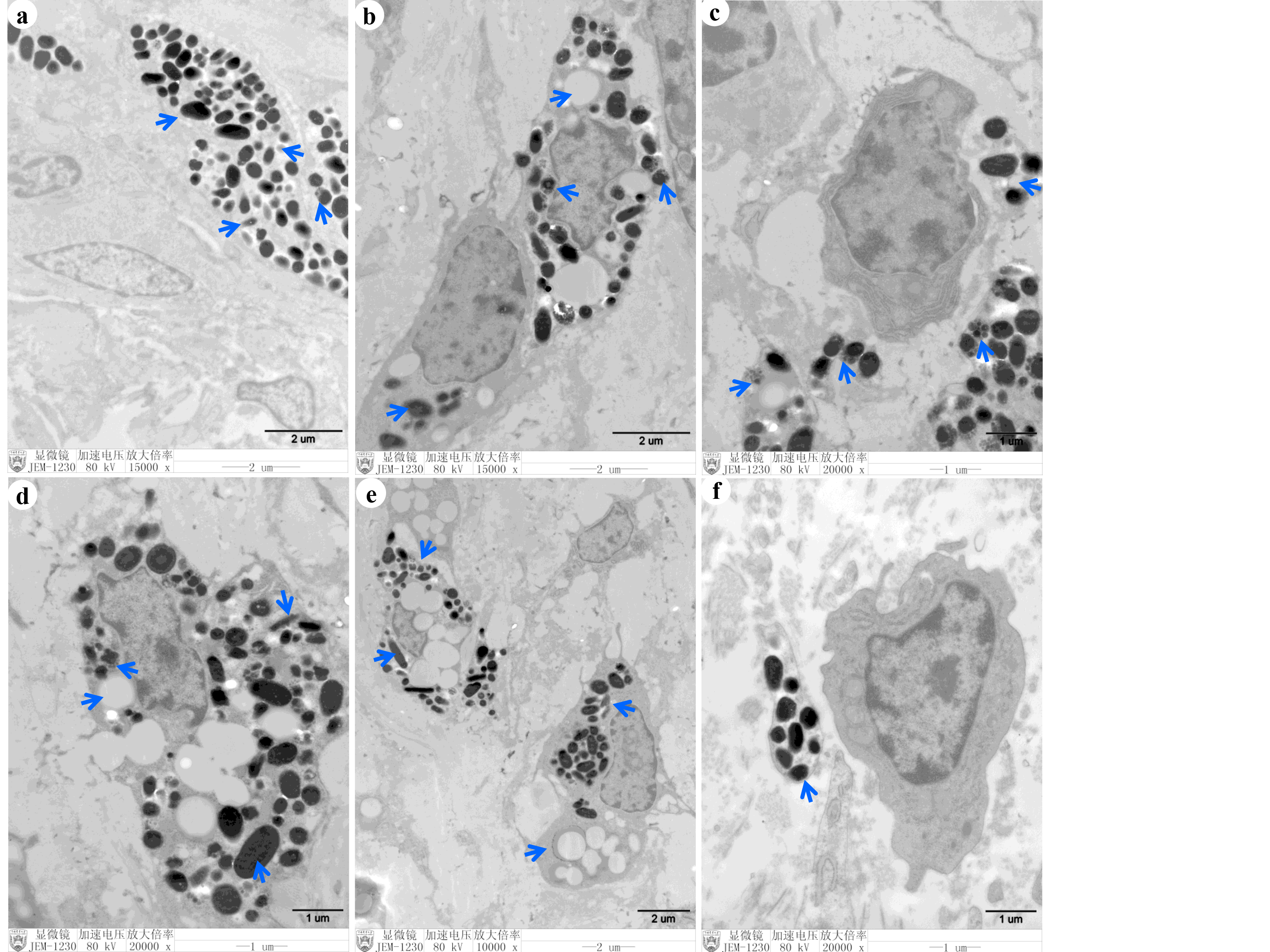

Supplement: S3 Fig — In 23-week-old SFs, melanosomes (blue arrow) at different stages of maturity (shown as rounds or ovals) were observed. (a) Lung. (b–f) Ovary. (TIF) [file pone.0125686.s003.tif]

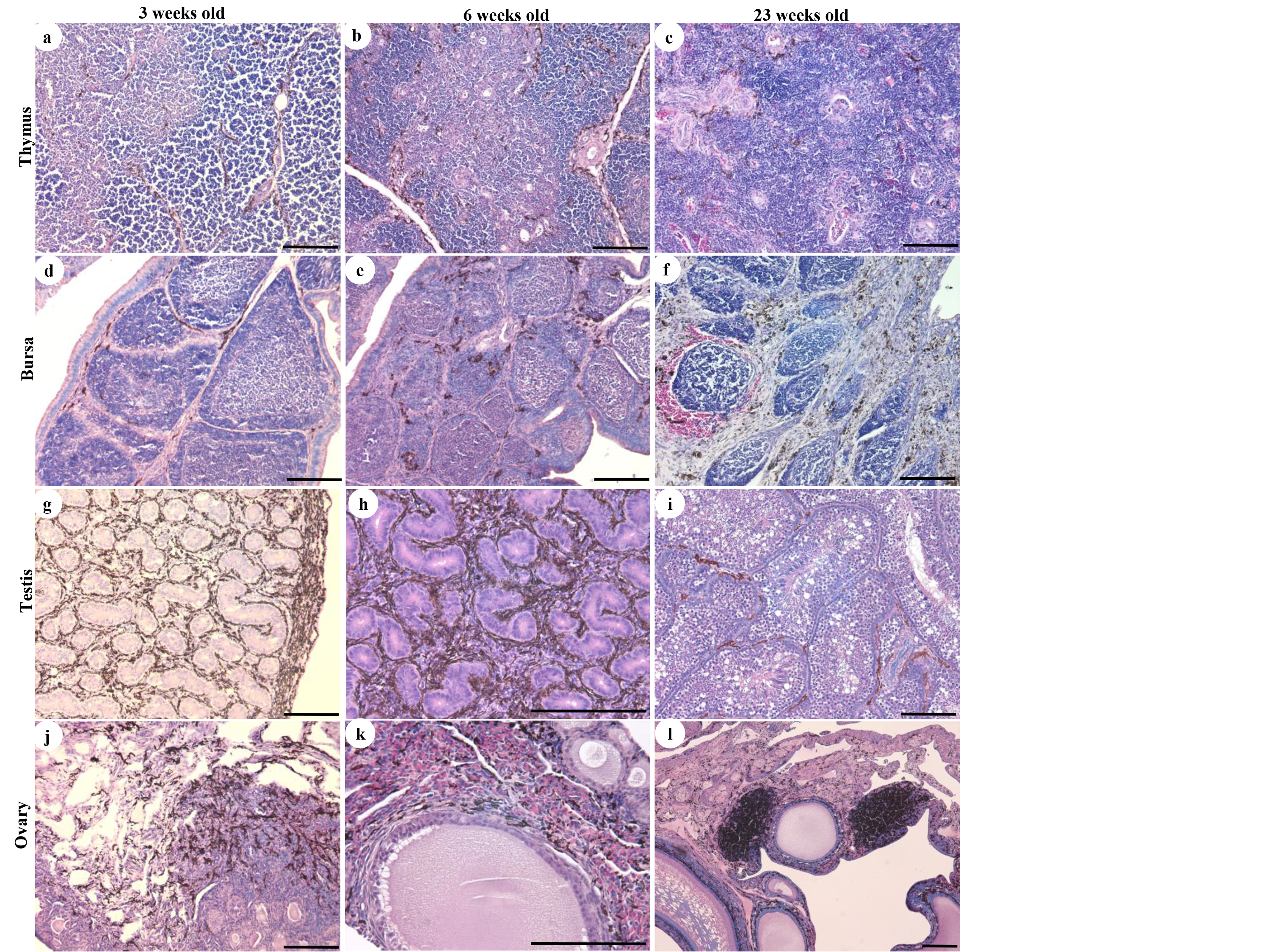

Supplement: S4 Fig — After hatching, the number of melanocytes increased, but no significant changes were observed after maturity. Scale bar = 100 μm. (TIF) [file pone.0125686.s004.tif]

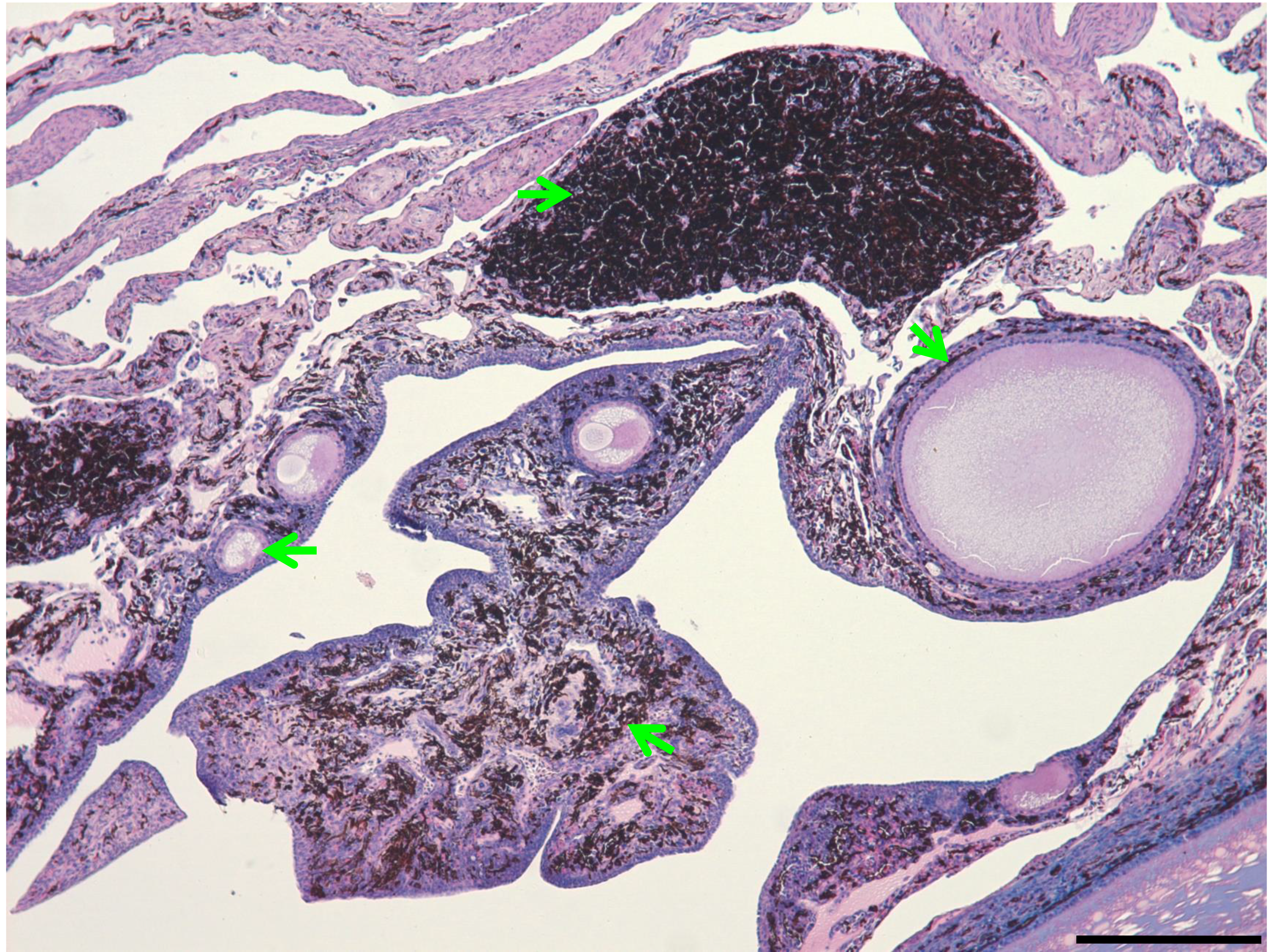

Supplement: S5 Fig — Melanocytes were observed in the secondary and mature follicles and in the corpus luteum, but not in primordial and primary follicles. Scale bar = 100 μm. (TIF) [file pone.0125686.s005.tif]

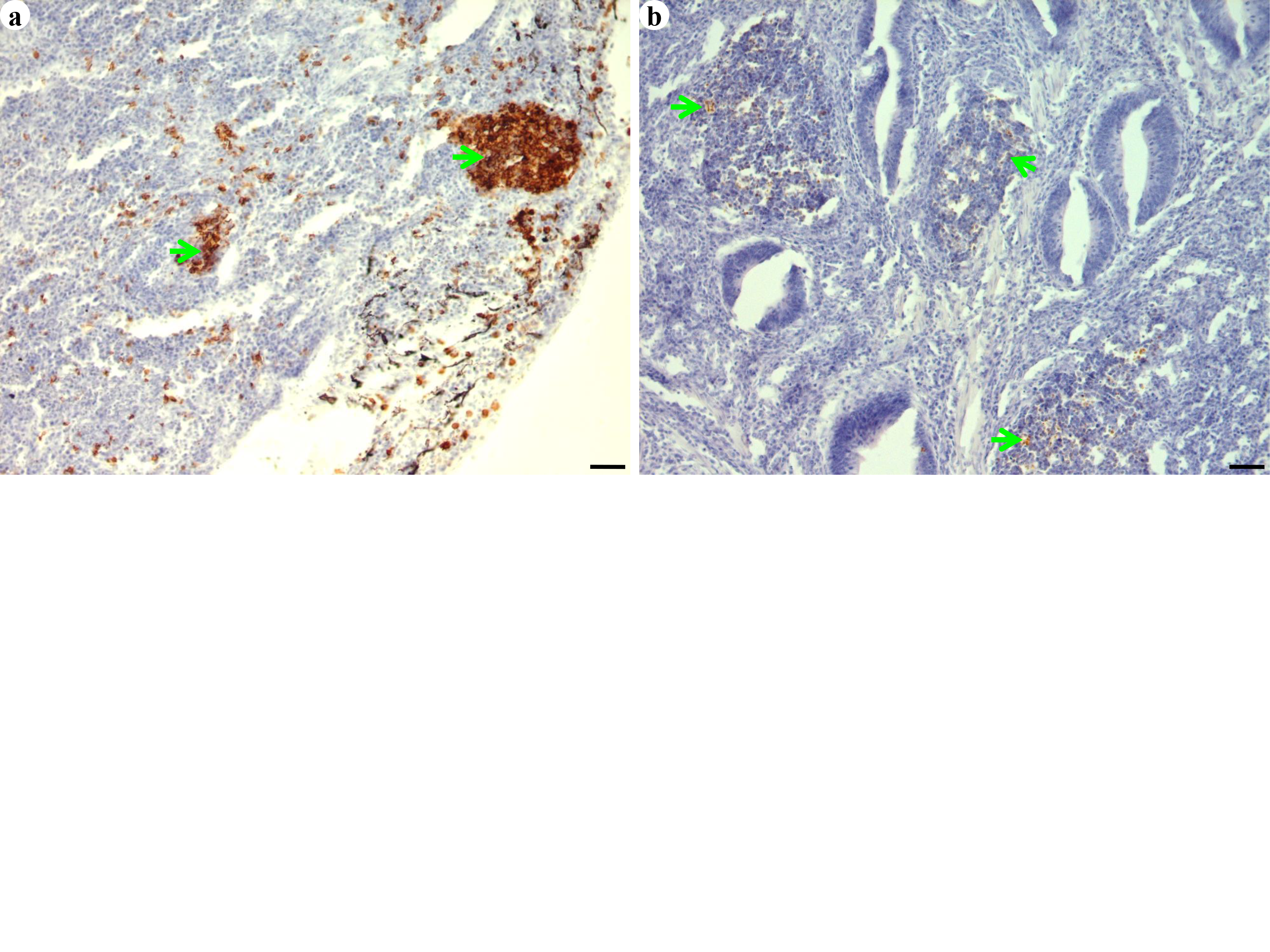

Supplement: S6 Fig — Lymphoid nodules with Bu-1+ cells in the spleen (a) and cecum (b). Scale bar = 100 μm. (TIF) [file pone.0125686.s006.tif]
